# Virulence gene polymorphisms in Shandong *Helicobacter pylori* strains and their relevance to gastric cancer

**DOI:** 10.1371/journal.pone.0309844

**Published:** 2024-09-09

**Authors:** Zhijing Xue, Weijia Li, Hailing Ding, Fengyan Pei, Jianzhong Zhang, Yanan Gong, Ruyue Fan, Fang Wang, Youjun Wang, Qing Chen, Yanran Li, Xinyu Yang, Yan Zheng, Guohai Su

**Affiliations:** 1 Department of Gastroenterology, Central Hospital Affiliated to Shandong First Medical University, Jinan, Shandong, China; 2 Research Center of Translational Medicine, Central Hospital Affiliated to Shandong First Medical University, Jinan, Shandong, China; 3 National Institute for Communicable Disease Control and Prevention, Chinese Center for Disease Control and Prevention, Beijing, China; 4 Department of Gastroenterology, Qilu Hospital of Shandong University, Jinan, Shandong, China; 5 The Faculty of Medicine, Qilu Institute of Technology, Jinan, Shandong, China; 6 Medical Research & Laboratory Diagnostic Center, Central Hospital Affiliated to Shandong First Medical University, Jinan, Shandong, China; 7 Shandong Center for Disease Control and Prevention, Jinan, Shandong, China; 8 Department of Cardiovascular Medicine, Central Hospital Affiliated to Shandong First Medical University, Jinan, Shandong, China; University of Manitoba, CANADA

## Abstract

**Background:**

*Helicobacter pylori* (*H*. *pylori*) virulence factors, particularly the *cagA* and *vacA* genotypes, play important roles in the pathogenic process of gastrointestinal disease.

**Methods:**

The *cagA* and *vacA* genotypes of 87 *H*. *pylori* strains were determined by PCR and sequencing. The EPIYA and CM motif patterns were analyzed and related to clinical outcomes. We examined the associations between the virulence genes of *H*. *pylori* and gastrointestinal diseases in Shandong, and the results were analyzed via the chi-square test and logistic regression model.

**Results:**

Overall, 76 (87.36%) of the strains carried the East Asian-type CagA, with the ABD types being the most prevalent (90.79%). However, no significant differences were observed among the different clinical outcomes. The analysis of CagA sequence types revealed 8 distinct types, encompassing 250 EPIYA motifs, including 4 types of EPIYA or EPIYA-like sequences. Additionally, 28 CM motifs were identified, with the most prevalent patterns being E (66.67%), D (16.09%), and W-W (5.75%). Notably, a significant association was discovered between strains with GC and the CM motif pattern D (*P* < 0.01). With respect to the *vacA* genotypes, the strains were identified as s1, s2, m1, m2, i1, i2, d1, d2, c1, and c2 in 87 (100%), 0 (0), 26 (29.89%), 61 (70.11%), 73 (83.91%), 14 (16.09%), 76 (87.36%), 11 (12.64%), 18 (20.69%), and 69 (79.31%), respectively. Specifically, the *vacA* m1 and c1 genotypes presented a significantly greater prevalence in strains from GC compared to CG (*P* < 0.05). Following adjustment for age and sex, the *vacA* c1 genotype demonstrated a notable association with GC (OR = 5.174; 95% CI, 1.402–20.810; *P* = 0.012). This association was both independent of and more pronounced than the correlations between *vacA* m1 and GC.

**Conclusions:**

CagA proteins possessing CM motif pattern D were more frequently observed in patients with GC (*P* < 0.01), implying a potentially higher virulence of CM motif pattern D than the other CM motif patterns. Moreover, a strong positive association was identified between the *vacA* c1 genotype and GC, indicating that the *vacA* c1 genotype is a robust risk indicator for GC among male patients aged ≥55 years in Shandong.

## Introduction

*Helicobacter pylori* (*H*. *pylori*) is a gram-negative bacterium known for its colonization of the human stomach and its significant role as a primary contributor to severe gastroduodenal diseases, such as chronic gastritis (CG), peptic ulcer disease (PUD), gastric adenocarcinoma (GAC), and mucosal-associated lymphoid tissue (MALT) lymphoma [1, 2]. Gastric cancer (GC) is the third most common cause of cancer-related mortality and ranks fifth among malignant tumors globally [3]. Although *H*. *pylori* infection is a crucial risk factor for the development of GC, only a minority of individuals infected with *H*. *pylori* progress to this severe condition [4]. In addition to genetic susceptibility, duration of infection, and environmental and lifestyle factors, an important reason for the different clinical outcomes of *H*. *pylori* infection is the virulence factors of *H*. *pylori* [5]. *H*. *pylori* can produce a variety of virulence factors, of which cytotoxin-associated gene A (CagA) and vacuolating cytotoxin (VacA) are the most extensively studied [6, 7].

CagA is one of the most important virulence factors of *H*. *pylori* related to the pathogenic mechanisms of GC and is encoded by the *cagA* gene located at the end of the *cag* pathogenicity island (*cag* PAI) [8, 9]. Research indicates that CagA-positive strains can cause more severe gastric mucosal damage and inflammatory responses, thereby substantially increasing the risk of developing PUD and GC [10, 11]. Notably, there is a regional disparity in CagA prevalence, with nearly all strains in East Asian countries being CagA-positive compared with approximately 50% in Western countries [12]. CagA is subdivided into the East Asian-type and Western-type on the basis of differences in the repeat sequences at its C-terminus, which contains tyrosine phosphorylation site EPIYA (Glu-Pro-Ile-Tyr-Ala) motifs [13, 14]. In accordance with the sequences surrounding the EPIYA motifs, four distinct EPIYA segments, EPIYA-A, EPIYA-B, EPIYA-C, and EPIYA-D, have been identified [15, 16]. Almost all the CagA-positive strains have EPIYA-A or EPIYA-B segments. The EPIYA-C segment is unique to the Western-type CagA, whereas the EPIYA-D segment characterizes the East Asian-type CagA [17, 18]. These differences in CagA contribute to variations in the pathogenicity of *H*. *pylori* strains in different regions. Compared with the Western-type CagA, the East Asian-type CagA with the EPIYA-D segment has a stronger binding affinity for src homology 2 (SH2)-containing protein tyrosine phosphatases (SHP-2). In addition, the Western-type CagA, which contains multiple EPIYA-C segments, has heightened SHP-2 binding activity, resulting in more pronounced cellular morphological changes [19]. The structural arrangement of the EPIYA segment sequences within CagA displays considerable diversity among strains. For example, over 60% of the Western-type CagA comprises the ABC type (66.5%), followed by the ABCC (20.3%) and ABCCC (4%) types. Conversely, the predominant type within the East Asian-type CagA is ABD (83.6%), followed by the ABBD, ABDD, and AABD types. A small proportion of Western-type and East Asian-type CagA exhibit more complex arrangements of EPIYA segment sequences [20, 21]. These variations in EPIYA repeat sequences play crucial roles in determining the structural polymorphism of the CagA protein, thereby influencing its functional diversity.

There is another motif composed of 16 amino acid residues, known as the CagA multimerization (CM) motif at the C-terminus of CagA [22, 23]. Western CM sequences (FPLKRHDKVDDLSKVG) are classified as typical Western CM (W-CM) motifs because they are similar to those obtained from patients in Western countries, whereas East Asian CM sequences (FPLRRSAAVNDLSKVG) are classified as typical East Asian CM (E-CM) motifs that are similar to those obtained from East Asian countries [24, 25]. Variations in positions 4, 6, 7, 8, and 10 (FPLxRxxxVxDLSKVG) between the W-CM and E-CM motifs were elucidated in a previous study [26]. The Western-type CagA is characterized by at least two CM sequences, whose number corresponds to the number of EPIYA-C segments, whereas the East Asian-type CagA exclusively harbors a single CM motif at the C-terminus [27]. The CM motif can mediate CagA dimerization and stabilize its binding to SHP-2 [22]. Additionally, CM motifs can also bind to polarity regulatory kinase partitioning defective 1/microtubule affinity regulating kinase (PAR1/MARK), consequently inhibiting the activity of kinases [28]. This multifaceted interaction underscores the intricate regulatory mechanisms involving the CagA protein and its CM motifs in the context of *H*. *pylori* infection.

VacA, which is encoded by vacuolating cytotoxin gene A (*vacA*), is another extensively studied virulence factor of *H*. *pylori* that can be endocytosed by host cells and induce vacuolation and various cellular activities, leading to cell death and cell membrane receptor binding [29, 30]. The *vacA* gene exhibits five polymorphic regions, denoted as signal (s)-, intermediate (i)-, middle (m)-, deletion (d)-, and central (c)-regions, fostering the production of VacA with varying toxicities in different strains [31]. On the basis of *vacA* allelic diversity in these regions, two different genotypes have been described for each region: s1 and s2 for the s-region, i1 and i2 for the i-region, m1 and m2 for the m-region, d1 and d2 for the d-region, and c1 and c2 for the c-region [30, 31]. The different combinations of *vacA* s-, m-, i-, d-, and c-region genotypes determine the vacuolating activity of various *H*. *pylori* strains [32–35]. Previous investigations have revealed that strains with the s1m1 genotype exhibit greater cytotoxicity than those with the s1m2 and s2m2 variants do, thereby increasing the risk of PUD or GC development [36]. Additionally, strains bearing the i1 genotype demonstrate a robust association with GC and heightened vacuolating cytotoxin activity [37, 38]. This nuanced understanding highlights the critical role of *vacA* and its genotypic variants in influencing the virulence potential of *H*. *pylori* strains.

Shandong Province, situated in eastern China along the Yellow Sea, has a substantial prevalence of *H*. *pylori* infection, reaching 83.15% (74/89), alongside an incidence of GC reported at 34.58 per 100,000 persons [39, 40]. Despite the high prevalence of *H*. *pylori* in Shandong, little is known about the local prevalence of the *cagA* and *vacA* genotypes, as well as their associations with gastroduodenal diseases. Thus, this study aimed to assess the prevalence of different genotypes of *cagA* and *vacA* and to determine the relationships between these genotypes and clinical outcomes via gene sequencing methods. This study provides valuable insights into the prevalence and genetic characteristics of *H*. *pylori* strains in Shandong, shedding light on their potential implications for the development of gastroduodenal diseases.

## Materials and methods

### Patients and gastric biopsies

The *H*. *pylori* infection study was conducted from December 2014 to December 2015 in Shandong. A total of 305 patients were enrolled in this study, including 107 females (age range: 20–73 years; mean age: 51.71 ± 11.68 years) and 198 males (age range: 23–78 years; mean age: 56.03 ± 10.55 years). Among them, 206 patients were from Central Hospital Affiliated with Shandong First Medical University (Jinan, Shandong Province, China), and 99 patients were from Rushan People’s Hospital (Weihai, Shandong Province, China). The inclusion criteria included patients with dyspeptic symptoms who underwent upper gastrointestinal tract endoscopy and patients who provided informed consent. The exclusions criteria included patients who had received any antibiotics at least 1 month prior to endoscopy, patients who had received nonsteroidal anti-inflammatory drugs, steroids or proton pump inhibitors at least 3 months prior to endoscopy, and patients who had received anti-*H*. *pylori* eradication treatment. The final endoscopy diagnoses were CG in 266 (87.21%) patients, GC in 10 (3.28%), and PUD in 29 (9.51%) including GU (22, 7.21%) and DU (7, 2.30%). Three biopsy samples were taken from the antrum of each patient for *H*. *pylori* culture and histological examination. The biopsy samples for culture were immediately stored in brain heart infusion broth (BHI, CM1135, Oxoid) containing 20% glycerin. Written informed consent was obtained from all participants under a protocol approved by the Ethical Committee of the National Institute for Communicable Disease Control and Prevention, Chinese Center for Disease Control and Prevention (Approval No. ICDC-2013001).

### *H*. *pylori* isolation and identification

The gastric biopsy samples were homogenized and cultured on the surface of Karmali agar (CM0935, Oxoid) plates supplemented with 5% defibrinated sheep blood or *H*. *pylori* selective supplement (vancomycin 10 μg/mL, trimethoprim 5 μg/mL, cefsulodin 5 μg/mL, and amphotericin B 5 μg/mL). The plates were incubated at 37°C under a microaerobic atmosphere (5% O_2_, 10% CO_2_, and 85% N_2_) for up to 10 days. *H*. *pylori* isolates were identified on the basis of colony morphology (smooth and translucent), gram staining (gram-negative with spiral-shaped bacilli), and positive reactions for oxidase, catalase, and urease. After subculturing on Karmali agar supplemented with 5% defibrinated sheep blood, all the isolates were stored at -80°C in BHI broth containing 20% glycerin.

### DNA extraction and genotyping

Bacterial DNA was extracted via the TIANamp Bacteria DNA Kit (TIANGEN, China) according to the manufacturer’s protocol. The *cagA* status was determined by polymerase chain reaction (PCR) amplification and direct sequencing using forward (5’-TGCGTGTGTGGCTGTTAGTAG-3’) and reverse (5’-CCCTAGTCGGTAATGGGTTGT-3’) primers designed in the 3’ repeat region of *cagA*, as described previously [41]. The presence of *cagA* was confirmed by primers for the conserved region of *cagA*: forward (5’-AGC AAAAAGCGACCTTGAAA-3’) and reverse (5’-AGTGGCTCAAGCTCGTGAAT-3’), as described previously [42]. The PCR conditions were denaturation for 5 min at 94°C, 35 cycles (94°C for 30 s, 54°C for 30 s, and 72°C for 40 s), and a final extension of 10 min at 72°C. The PCR products were purified via the E.Z.N.A.® Gel Extraction Kit (OMEGA, USA) according to the manufacturer’s instructions, and the *cagA* genotype (East Asian-type or Western-type) was confirmed by sequencing. The nucleotide sequences of the *cagA* 3’ variable region were subjected to translation via BioEdit version 7.2.5. The EPIYA segment types and CM motif of CagA were compared using the program WebLogo 3 (http://weblogo.three.plusone.com/).

Genotyping of the *vacA* s- (s1 or s2), m- (m1 or m2), i- (i1 or i2), d- (d1 or d2), and c- (c1 or c2) regions was performed following previously described methods [43–46]. Multiple sequence alignments of the *vacA* sequences were generated via MAFFT 7 (http://mafft.cbrc.jp/alignment/server/). The genotyping of the s- and m-regions of *vacA* was performed according to the method of Atherton et al. [43, 46], the i- and d-regions were typed according to the method of Ogiwara et al. [44], and the c-region was determined via the method of Bakhti et al. [45].

### Statistical analysis

Statistical analyses were conducted via SPSS statistical software version 20 (SPSS, Chicago, USA). The chi-square (χ^2^) test and Fisher’s exact test were used to assess the associations between each genotype and different regions, as well as clinical outcomes. A logistic regression model was used to evaluate the relationships between the candidate genes and clinical outcomes. The odds ratio (OR) and 95% confidence interval (CI) were obtained for multivariate analysis and used to estimate the risk. *P* < 0.05 was considered statistically significant.

## Results

### Patient characteristics

A total of 87 *H*. *pylori* strains (87/305, 28.52%) were successfully isolated from dyspeptic patients, consisting of 62 males (age range: 32–78 years; mean age: 58.06 ± 8.53 years) and 25 females (age range: 36–73 years; mean age: 54.84 ± 9.59 years). Among these strains, 67 strains were isolated from subjects with CG, 10 from PUD patients (4 with GU and 6 with DU), and 10 from GC patients. Stratified analyses were conducted on the basis of sex distribution: females (25/87, 28.74%) and males (62/87, 71.26%), as well as two age categories: patients aged <55 years (37/87, 42.53%) and those aged ≥55 years (50/87, 57.47%). Within the CG group, further classification identified chronic superficial gastritis (CSG) in 16 patients (23.88%) and chronic atrophic gastritis (CAG) in 51 patients (76.12%) based on the presence or absence of glandular atrophy. Among the GC patients, all individuals were aged over 55 years, with 80% being male. Statistical analyses revealed a significant association between age and GC (*P* = 0.007), whereas no statistically significant difference was detected between sex and GC (*P* = 0.260) (Table 1).

**Table 1:** Characteristics of patients enrolled in this study.

Types of diseases	No. of patients (%)	Age groups		Sex groups	
		No. of ≥55 (%)	No. of <55 (%)	No. of males (%)	No. of females (%)
CG	67/87 (77.01)	33/67 (49.25)	34/67 (50.75)	49/67 (73.13)	18/67 (26.87)
CSG	16/67 (23.88)	7/16 (43.75)	9/16 (56.25)	10/16 (62.5)	6/16 (37.5)
CAG	51/67 (76.12)	26/51 (50.98)	25/51 (49.02)	39/51 (76.47)	12/51 (23.53)
PUD	10/87 (11.49)	7/10 (70)	3/10 (30)	5/10 (50)	5/10 (50)
GU	4/10 (40)	3/4 (75)	1/4 (25)	1/4 (25)	3/4 (75)
DU	6/10 (60)	4/6 (66.67)	2/6 (33.33)	4/6 (66.67)	2/6 (33.33)
GC	10/87 (11.49)	**10/10 (100)** ^**a**^	0 (0)	8/10 (80)	2/10 (20)
Total	87/87 (100)	50/87 (57.47)	37/87 (42.53)	62/87 (71.26)	25/87 (28.74)

CG: chronic gastritis; CSG: chronic superficial gastritis; CAG: chronic atrophic gastritis; PUD: peptic ulcer disease; GU: gastric ulcer; DU: duodenal ulcer; GC: gastric cancer. ^a^ Boldface data indicate a significant difference.

### The prevalence of *H*. *pylori cagA* and *vacA* genotypes

All strains tested were successfully detected for the *cagA* and *vacA* genotypes. Among these strains, 62 strains were isolated from Jinan (53 with CG and 9 with PUD), and 25 were isolated from Weihai (14 with CG, 1 with PUD, and 10 with GC). As shown in S1 Table, we analyzed the distribution of *cagA* and *vacA* genotypes based on age, gender, and diseases. Statistical analysis showed no significant association between different genotypes and age and gender, and no statistically significant difference was observed between different genotypes in GSC and GAC. The distributions of the *cagA* and *vacA* genotypes in Shandong are summarized in Table 2. The East Asian-type *cagA* was predominant, constituting 87.36% (76/87) of the strains, whereas the Western-type *cagA* was identified in 12.64% (11/87) of the cases. The prevalence of the East Asian-type *cagA* was 85.48% in Jinan and 92% in Weihai, whereas that of the Western-type *cagA* was present in 14.52% and 8% of strains from Jinan and Weihai, respectively. Statistical analysis revealed no significant differences in the distribution of the *cagA* genotypes between the two distinct regions (χ^2^ = 0.685, *P* > 0.05).

**Table 2:** Prevalence of *H*. *pylori cagA and vacA* genotypes in Shandong.

Genotypes	No. of isolates		
	Jinan (n = 62)	Weihai (n = 25)	Total (n = 87)
*cagA*	62 (100)	25 (100)	87 (100)
East Asian-type *cagA*	53 (85.48)	23 (92)	76 (87.36)
Western-type *cagA*	9 (14.52)	2 (8)	11 (12.64)
*vacA*	62 (100)	25 (100)	87 (100)
s1	62 (100)	25 (100)	87 (100)
s2	0 (0)	0 (0)	0 (0)
m1	15 (24.19)	11 (44)	26 (29.89)
m2	47 (75.81)	14 (56)	61 (70.11)
i1	52 (83.87)	21 (84)	73 (83.91)
i2	10 (16.13)	4 (16)	14 (16.09)
d1	54 (87.10)	22 (88)	76 (87.36)
d2	8 (12.90)	3 (12)	11 (12.64)
c1	10 (16.13)	8 (32)	18 (20.69)
c2	52 (83.87)	17 (68)	69 (79.31)
s1m1i1	15 (24.19)	11 (44)	26 (29.89)
s1m1d1	15 (24.19)	11 (44)	26 (29.89)
s1m1c1	10 (16.13)	8 (32)	18 (20.69)
s1m1i2	0 (0)	0 (0)	0 (0)
s1m1d2	0 (0)	0 (0)	0 (0)
s1m1c2	5 (8.06)	3 (12)	8 (9.20)
s1m2i1	37 (59.68)	10 (40)	47 (54.02)
s1m2d1	39 (62.9)	11 (44)	50 (57.47)
s1m2c1	0 (0)	0 (0)	0 (0)
s1m2i2	10 (16.13)	4 (16)	14 (16.09)
s1m2d2	8 (12.9)	3 (12)	11 (12.64)
s1m2c2	47 (75.81)	14 (56)	61 (70.11)
s1m2i1d1c2	35 (56.45)	9 (36)	44 (50.57)
s1m1i1d1c1	10 (16.13)	8 (32)	18 (20.69)
s1m1i1d1c2	5 (8.06)	3 (12)	8 (9.20)
s1m2i2d2c2	6 (9.68)	2 (8)	8 (9.20)
s1m2i2d1c2	4 (6.45)	2 (8)	6 (6.90)
s1m2i1d2c2	2 (3.23)	1 (4)	3 (3.45)

Values in parentheses are percentages.

The *vacA* genotypes were evaluated based on the five polymorphic regions: the s-, m-, i-, d-, and c-regions. All strains were successfully identified by their *vacA* genotypes, which were classified as s1, s2, m1, m2, i1, i2, d1, d2, c1, and c2 in 87 (100%), 0 (0), 26 (29.89%), 61 (70.11%), 73 (83.91%), 14 (16.09%), 76 (87.36%), 11 (12.64%), 18 (20.69%), and 69 (79.31%) strains, respectively (Table 2). Statistical analysis revealed no significant differences in the *vacA* genotypes between the two different regions (*P* > 0.05). For the combination of the *vacA* s-, m-, i-, d-, and c-regions, 44 strains (50.57%) presented s1m2i1d1c2, followed by 18 strains (20.69%) with s1m1i1d1c1, 8 strains (9.20%) with s1m1i1d1c2, 8 strains (9.20%) with s1m2i2d2c2, 6 strains (6.90%) with s1m2i2d1c2, and 3 strains (3.45%) with s1m2i1d2c2. The two most common *vacA* genotype combinations were s1m2i1d1c2 and s1m1i1d1c1 in the two different regions, but the differences were not statistically significant (*P* > 0.05). An examination of the two different regions of the *vacA* genotype revealed that all strains possessing s1 and m1 also carried i1 and d1 (26/87, 29.89%). Conversely, strains containing the s1 and m2 genotypes predominantly harbored the c2 genotype (68.97%, 60/87). Furthermore, the investigation revealed the presence of every type of i-region and d-region among strains characterized by the s1 and m2 *vacA* genotypes (Table 2).

### Associations between virulence factors and clinical outcomes

As shown in Table 3, the East Asian-type *cagA* was present in 88.06% of isolates from CG patients, 80% from PUD patients, and 90% from GC patients, whereas the Western-type *cagA* was identified in 11.94%, 20%, and 10%, respectively. In this study, there was no significant difference between the East Asian-type or Western-type *cagA* genotypes and clinical outcomes (*P* > 0.05). A total of 13.21% of GC patients were infected with Western-type *cagA* strains in Jinan, which was higher than that in Weihai (7.14%); however, the difference was not statistically significant (*P* > 0.05).

**Table 3:** Association between *H*. *pylori* virulence factors and clinical outcomes.

Genotypes	No. of isolates								
	Jinan (n = 62)			Weihai (n = 25)			Total (n = 87)		
	CG (53)	PUD (9)	GC (0)	CG (14)	PUD (1)	GC (10)	CG (67)	PUD (10)	GC (10)
*cagA*									
East Asian-type *cagA*	46 (86.79)	7 (77.78)	0 (0)	13 (92.86)	1 (100)	9 (90)	59 (88.06)	8 (80)	9 (90)
Western-type *cagA*	7 (13.21)	2 (22.22)	0 (0)	1 (7.14)	0(0)	1 (10)	8 (11.94)	2 (20)	1 (10)
*vacA*			0 (0)						
s1	53 (100)	9 (100)	0 (0)	14 (100)	1 (100)	10 (100)	67 (100)	10 (100)	10 (100)
s2	0 (0)	0 (0)	0 (0)	0 (0)	0 (0)	0 (0)	0 (0)	0 (0)	0 (0)
m1	14 (26.42)	1 (11.11)	0 (0)	4 (28.57)	1 (100)	6 (60)	18 (26.87)	2 (20)	**6 (60)** ^a^
m2	39 (73.58)	8 (88.89)	0 (0)	10 (71.43)	0 (0)	4 (40)	49 (73.13)	8 (80)	4 (40)
i1	45 (84.91)	7 (77.78)	0 (0)	12 (85.71)	1(100)	8 (80)	57 (85.07)	8 (80)	8 (80)
i2	8 (15.09)	2 (22.22)	0 (0)	2 (14.29)	0 (0)	2 (20)	10 (14.93)	2 (20)	2 (20)
d1	46 (86.79)	8 (88.89)	0 (0)	12 (85.71)	1 (100)	9 (90)	58 (86.57)	9 (90)	9 (90)
d2	7 (13.21)	1 (11.11)	0 (0)	2 (14.29)	0 (0)	1 (10)	9 (13.43)	1 (10)	1 (10)
c1	10 (18.87)	0 (0)	0 (0)	3 (21.43)	0 (0)	5 (50)	13 (19.40)	0 (0)	**5 (50)** ^a^
c2	43 (81.13)	9 (100)	0 (0)	11 (78.57)	1 (100)	5 (50)	54 (80.60)	10 (100)	5 (50)
m1i1	14 (26.42)	1 (11.11)	0 (0)	4 (28.57)	1 (100)	6 (60)	18 (26.87)	2 (20)	**6 (60)** ^a^
m2i2	8 (15.09)	2 (22.22)	0 (0)	2 (14.29)	0 (0)	2 (20)	10 (14.93)	2 (20)	2 (20)
m1d1	14 (26.42)	1 (11.11)	0 (0)	4 (28.57)	1 (100)	6 (60)	18 (26.87)	2 (20)	**6 (60)** ^a^
m2d2	7 (13.21)	1 (11.11)	0 (0)	2 (14.29)	0 (0)	1 (10)	9 (13.43)	1 (10)	1 (10)
m1c1	10 (18.87)	0 (0)	0 (0)	3 (21.43)	0 (0)	5 (50)	13 (19.40)	0 (0)	**5 (50)** ^a^
m2c2	39 (73.58)	8 (88.89)	0 (0)	10 (71.43)	0 (0)	4 (40)	49 (73.13)	8 (80)	4 (40)
i1d1	43 (81.13)	7 (77.78)	0 (0)	11 (78.57)	1 (100)	8 (80)	54 (80.60)	8 (80)	8 (80)
i2d2	5 (9.43)	1 (11.11)	0 (0)	1 (7.14)	0 (0)	1 (10)	6 (8.96)	1 (10)	1 (10)
i1c1	10 (18.87)	0 (0)	0 (0)	3 (21.43)	0 (0)	5 (50)	13 (19.40)	0 (0)	**5 (50)** ^a^
i2c2	8 (15.09)	2 (22.22)	0 (0)	2 (14.29)	0 (0)	2 (20)	10 (14.93)	4 (40)	0 (0)
d1c1	10 (18.87)	0 (0)	0 (0)	3 (21.43)	0 (0)	5 (50)	13 (19.40)	0 (0)	**5 (50)** ^a^
d2c2	7 (13.21)	1 (11.11)	0 (0)	2 (14.29)	0 (0)	1 (10)	9 (13.43)	1 (10)	1 (10)
m1i1d1	14 (26.42)	1 (11.11)	0 (0)	4 (28.57)	1 (100)	6 (60)	18 (26.87)	2 (20)	**6 (60)** ^a^
m2i2d2	5 (9.43)	1 (11.11)	0 (0)	1 (7.14)	0 (0)	1 (10)	6 (8.96)	1 (10)	1 (10)
m1i1c1	10 (18.87)	0 (0)	0 (0)	3 (21.43)	0 (0)	5 (50)	13 (19.40)	0 (0)	**5 (50)** ^a^
m2i2c2	8 (15.09)	2 (22.22)	0 (0)	2 (14.29)	0 (0)	2 (20)	10 (14.93)	2 (20)	2 (20)
m1d1c1	10 (18.87)	0 (0)	0 (0)	3 (21.43)	0 (0)	5 (50)	13 (19.40)	0 (0)	**5 (50)** ^a^
m2d2c2	7 (13.21)	1 (11.11)	0 (0)	2 (14.29)	0 (0)	1 (10)	9 (13.43)	1 (10)	1 (10)
i1d1c1	10 (18.87)	0 (0)	0 (0)	3 (21.43)	0 (0)	5 (50)	13 (19.40)	0 (0)	**5 (50)** ^a^
i2d2c2	5 (9.43)	1 (11.11)	0 (0)	1 (7.14)	0 (0)	1 (10)	6 (8.96)	1 (10)	1 (10)
m1i1d1c1	10 (18.87)	0 (0)	0 (0)	3 (21.43)	0 (0)	5 (50)	13 (19.40)	0 (0)	**5 (50)** ^a^
m2i2d2c2	5 (9.43)	1 (11.11)	0 (0)	1 (7.14)	0 (0)	1 (10)	6 (8.96)	1 (10)	1 (10)
s1m2i1d1c2	29 (54.72)	6 (66.67)	0 (0)	7 (50)	0 (0)	2 (20)	36 (53.73)	6 (60)	2 (20)
s1m1i1d1c1	10 (18.87)	0 (0)	0 (0)	3 (21.43)	0 (0)	5 (50)	13 (19.40)	0 (0)	**5 (50)** ^a^
s1m1i1d1c2	4 (7.55)	1 (11.11)	0 (0)	1 (7.14)	1 (100)	1 (10)	5 (7.46)	2 (20)	1 (10)
s1m2i2d2c2	5 (9.43)	1 (11.11)	0 (0)	1 (7.14)	0 (0)	1 (10)	6 (8.96)	1 (10)	1 (10)
s1m2i2d1c2	3 (5.66)	1 (11.11)	0 (0)	1 (7.14)	0 (0)	1 (7.14)	4 (5.97)	1 (10)	1 (10)
s1m2i1d2c2	2 (3.77)	0 (0)	0 (0)	1 (7.14)	0 (0)	0 (0)	3 (4.48)	0 (0)	0 (0)

CG: chronic gastritis; PUD: peptic ulcer disease; GC: gastric cancer. Values in parentheses are percentages. ^a^ Boldface data indicate a significant difference.

The prevalence of the *vacA* m1 genotype was significantly greater in strains from GC patients (60%) than in those from CG patients (26.87%) (χ^2^ = 5.472, *P* < 0.05; Table 3). The *vacA* c1 genotype was significantly more prevalent in strains from GC patients (50%) than in those from CG patients (19.40%) (χ^2^ = 5.915, *P* < 0.05). The results of the univariate analysis revealed that *vacA* m1 and c1 genotypes were positively correlated with GC and increased the risk of GC (OR = 4.275; 95% CI, 1.093–16.720; *P* = 0.027; and OR = 4.923; 95% CI, 1.244–19.482; *P* = 0.015, respectively). In contrast, the presence of *vacA* m2 and c2 genotypes was negatively correlated with GC and significantly decreased the risk of GC (OR = 0.234; 95% CI, 0.060–0.915; *P* = 0.027; and OR = 0.203; 95% CI, 0.051–0.804; *P* = 0.015, respectively). There was no significant association between the other *vacA* genotypes and different diseases (*P* > 0.05, Table 4). The presence of the *vacA* m1 and c1 genotypes in combination with the other genotypes further increased the risk of GC. The ORs for *vacA* m1i1 were 4.275 (95% CI, 1.093–16.720; *P* = 0.027), m1d1 4.275 (95% CI, 1.093–16.720; *P* = 0.027), m1c1 4.923 (95% CI, 1.244–19.482; *P* = 0.015), i1c1 4.923 (95% CI, 1.244–19.482; *P* = 0.015), d1c1 4.923 (95% CI, 1.244–19.482; *P* = 0.015), m1i1d1 4.275 (95% CI, 1.093–16.720; *P* = 0.027), m1i1c1 4.923 (95% CI, 1.244–19.482; *P* = 0.015), m1d1c1 4.923 (95% CI, 1.244–19.482; *P* = 0.015), i1d1c1 4.923 (95% CI, 1.244–19.482; *P* = 0.015), m1i1d1c1 4.923 (95% CI, 1.244–19.482; *P* = 0.015), and s1m1i1d1c1 4.923 (95% CI, 1.244–19.482; *P* = 0.015). The associations of the other *vacA* genotype combinations with clinical outcomes are shown in Table 4. We used multivariate analysis to examine the relative importance of the *vacA* m and c genotypes as risk factors for GC. The results demonstrated that the only factor with a significant adjusted OR was the *vacA* c1 genotype, and the OR was 5.174 (95% CI, 1.402–20.810; *P* = 0.012). The statistical analysis revealed a significantly greater frequency of the *vacA* c1 genotype in male patients with GC aged ≥55 years than in male patients with CG aged ≥55 years, with a multivariate analysis OR of 4.386 (95% CI, 1.341–19.248; *P* = 0.023).

**Table 4:** The relationship between *H*. *pylori vacA* genotypes and clinical outcomes.

Genotypes	CG			PUD			GC ^a^		
	OR	95%CI	P	OR	95%CI	P	OR	95%CI	P
m1	0.551	0.194–1.567	0.260	0.552	0.109–2.798	0.468	**4.275**	**1.093–16.720**	**0.027**
m2	1.815	0.638–5.160	0.260	1.811	0.357–9.178	0.468	0.234	0.060–0.915	0.027
i1	1.425	0.394–5.152	0.588	0.738	0.139–3.913	0.721	0.369	0.090–1.423	0.136
i2	0.702	0.194–2.537	0.588	1.345	0.256–7.175	0.721	2.708	0.703–10.437	0.136
d1	0.716	0.142–3.621	0.685	1.343	0.153–11.767	0.789	1.343	0.153–11.767	0.789
d2	1.397	0.276–7.063	0.685	0.744	0.085–6.521	0.789	0.744	0.085–6.521	0.789
c1	0.722	0.222–2.349	0.588	1.169	1.061–1.289	0.086	**4.923**	**1.244–19.482**	**0.015**
c2	1.385	0.426–4.503	0.588	0.855	0.776–0.942	0.086	0.203	0.051–0.804	0.015
m1i1	0.551	0.194–1.567	0.260	0.552	0.109–2.798	0.468	**4.275**	**1.093–16.720**	**0.027**
m2i2	0.702	0.194–2.537	0.588	1.345	0.256–7.175	0.721	1.354	0.256–7.175	0.721
m1d1	0.551	0.194–1.567	0.260	0.552	0.109–2.798	0.468	**4.275**	**1.093–16.720**	**0.027**
m2d2	1.397	0.276–7.063	0.685	0.744	0.085–6.521	0.789	0.744	0.085–6.521	0.789
m1c1	0.722	0.222–2.349	0.588	1.169	1.061–1.289	0.086	**4.923**	**1.244–19.482**	**0.015**
m2c2	1.185	0.638–5.160	0.260	1.811	0.357–9.178	0.468	0.234	0.060–0.915	0.027
i1d1	1.038	0.297–3.631	0.953	0.968	0.186–5.034	0.969	0.968	0.186–5.034	0.969
i2d2	0.885	0.164–4.771	0.887	1.111	0.122–10.101	0.925	1.111	0.122–10.101	0.925
i1c1	0.722	0.222–2.349	0.588	1.169	1.061–1.289	0.086	**4.923**	**1.244–19.482**	**0.015**
i2c2	0.702	0.194–2.537	0.588	1.050	0.954–1.155	0.656	1.159	1.058–1.270	0.141
d1c1	0.722	0.222–2.349	0.588	1.169	1.061–1.289	0.086	**4.923**	**1.244–19.482**	**0.015**
d2c2	1.397	0.276–7.063	0.685	0.744	0.085–6.521	0.789	0.744	0.085–6.521	0.789
m1i1d1	0.551	0.194–1.567	0.260	0.552	0.109–2.798	0.468	**4.275**	**1.093–16.720**	**0.027**
m2i2d2	0.885	0.164–4.771	0.887	1.111	0.122–10.101	0.925	1.111	0.122–10.101	0.925
m1i1c1	0.722	0.222–2.349	0.588	1.169	1.061–1.289	0.086	**4.923**	**1.244–19.482**	**0.015**
m2i2c2	0.702	0.194–2.537	0.588	1.345	0.256–7.175	0.721	1.354	0.256–7.175	0.721
m1d1c1	0.722	0.222–2.349	0.588	1.169	1.061–1.289	0.086	**4.923**	**1.244–19.482**	**0.015**
m2d2c2	1.397	0.276–7.063	0.685	0.744	0.085–6.521	0.789	0.744	0.085–6.521	0.789
i1d1c1	0.722	0.222–2.349	0.588	1.169	1.061–1.289	0.086	**4.923**	**1.244–19.482**	**0.015**
i2d2c2	0.885	0.164–4.771	0.887	1.111	0.122–10.101	0.925	1.111	0.122–10.101	0.925
m1i1d1c1	0.722	0.222–2.349	0.588	1.169	1.061–1.289	0.086	**4.923**	**1.244–19.482**	**0.015**
m2i2d2c2	0.885	0.164–4.771	0.887	1.111	0.122–10.101	0.925	1.111	0.122–10.101	0.925
s1m2i1d1c2	1.742	0.631–4.808	0.281	1.539	0.402–5.889	0.526	0.208	0.042–1.046	0.040
s1m1i1d1c1	0.722	0.222–2.349	0.588	1.169	1.061–1.289	0.086	**4.923**	**1.244–19.482**	**0.015**
s1m1i1d1c2	0.457	0.099–2.108	0.306	2.958	0.509–17.184	0.209	1.111	0.122–10.101	0.925
s1m2i2d2c2	0.885	0.164–4.771	0.887	1.111	0.122–10.101	0.925	1.111	0.122–10.101	0.925
s1m2i2d1c2	0.571	0.097–3.376	0.533	1.600	0.168–15.273	0.681	1.600	0.168–15.273	0.681
s1m2i1d2c2	1.313	1.165–1.479	0.336	1.135	1.049–1.228	0.525	1.135	1.049–1.228	0.525

CG: chronic gastritis; PUD: peptic ulcer disease; GC: gastric cancer. Values in parentheses are percentages. ^a^ Boldface data indicate a significant difference.

### CagA sequences and EPIYA segment types

In addition to the four major segments originally designated, we previously defined several rare segments, including EPIYA-B’, EPIYA-B”, and EPIYA-D’ [47]. A total of 8 sequence types were obtained from 87 CagA strains. The predominant CagA type was the East Asian-type, with 90.79% (69/76) of East Asian-type CagA strains displaying the typical ABD types, whereas only 3.95% (3/76) were ABD’. Only 11 strains carried the Western-type CagA, which included the AB, ABC, ABCC, and C subtypes. The distributions of the EPIYA segment types of CagA in various clinical outcomes are shown in Table 5. The prevalence of ABD was 90% (9/10) in GC patients, which was higher than the percentages reported in CG (79.10%) and PUD patients (70%). However, the difference was not significant (χ^2^ = 1.226, *P* > 0.05).

**Table 5:** Association between EPIYA segment types of CagA and clinical outcomes.

Type	No. of isolates			
	CG (67)	PUD (10)	GC (10)	Total (n = 87)
East Asian-type CagA				
ABD	53 (79.10)	7 (70)	9 (90)	69 (79.31)
ABD’	3 (4.48)	0 (0)	0 (0)	3 (4.84)
BD	1 (1.49)	0 (0)	0 (0)	1 (1.15)
D	2 (2.99)	1 (10)	0 (0)	3 (3.45)
Total	59 (88.06)	8 (80)	9 (90)	76 (87.36)
Western-type CagA				
AB*	1 (1.49)	0 (0)	0 (0)	1 (1.15)
ABC	5 (7.46)	2 (20)	0 (0)	7 (8.05)
ABCC	0 (0)	0 (0)	1 (10)	1 (1.15)
C	2 (3.77)	0 (0)	0 (0)	2 (2.30)
Total	8 (11.94)	2 (20)	1 (10)	11 (12.64)

CG: chronic gastritis; PUD: peptic ulcer disease; GC: gastric cancer. Values in parentheses are percentages. *AB type was defined as Western-type CagA according to the sequence of B segment (TGQVANLEEPIYTQVAKKVKAKIDRLNQIASGLGGVGQAAG for Western-type B segment and TGQVASPEEPIYAQVAKKVSAKIDQLNEATS for East Asian-type B segment).

**Fig 1:**
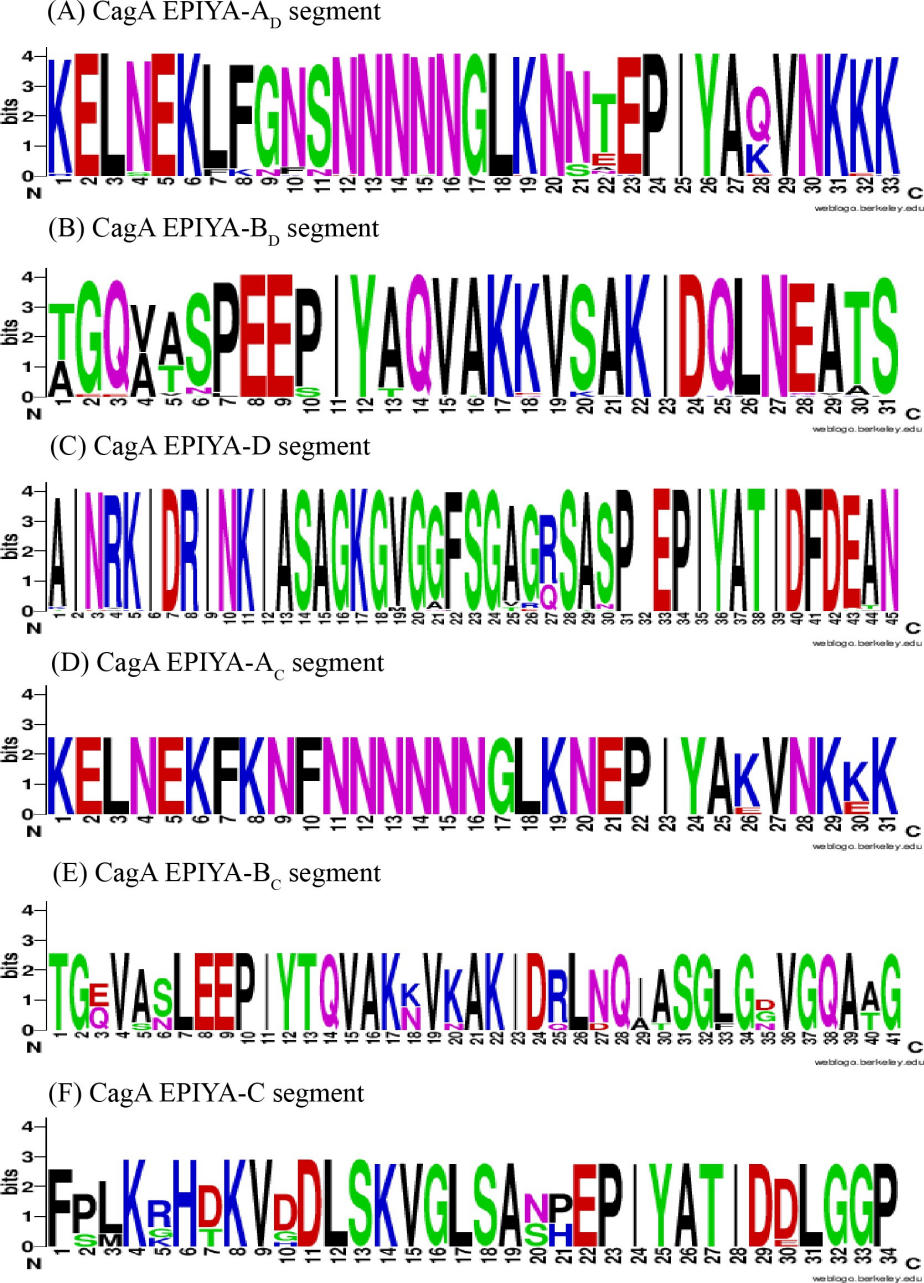
Variation in the CagA amino acid sequence of East Asian-type and Western-type CagA.

Through sequence alignment, variations in amino acid sequences were observed within the same segments, particularly in segments B_C_ and B_D_. Furthermore, the amino acid sequences of segments C and D displayed distinct differences when analyzed via WebLogo 3 (Fig 1). The EPIYA motifs in these strains were also evaluated (Table 6). A total of 250 EPIYA motifs were obtained from the 87 CagA strains, including 4 types of EPIYA or EPIYA-like sequences. The three most frequent EPIYA motifs were EPIYA (231/250 = 92.4%), EPIYT (5.2%), and ESIYA (2.16%), which was consistent with our previous study that examined 503 CagA strains deposited in GenBank [47]. The EPIYA-B motif displayed the greatest degree of variation in the five amino acids (e.g., EPIYA, EPIYT, and ESIYA), with EPIYT being more prevalent than EPIYA in the Western-type CagA strains (90% versus 10%).

**Table 6:** Frequencies of the four types of EPIYA motifs.

Type	A motif		B motif		C or D motif		All motifs	
	Motif	No.	Motif	No.	Motif	No.	Motif	No.
All CagA type	EPIYA	80	EPIYA	65	EPIYA	86	EPIYA	231
	KPIYA	1	EPIYT	13			EPIYT	13
			ESIYA	5			ESIYA	5
							KPIYA	1
Total		81		83				250
East Asian-type CagA	EPIYA	71	EPIYA	64	EPIYA	76	EPIYA	211
	KPIYA	1	EPIYT	4			EPIYT	4
			ESIYA	5			ESIYA	5
							KPIYA	1
Total		72		73		76		221
Western-type CagA	EPIYA	9	EPIYA	1	EPIYA	10	EPIYA	20
			EPIYT	9			EPIYT	9
Total		9		10		10		29

### CM motifs and patterns

**Fig 2:**
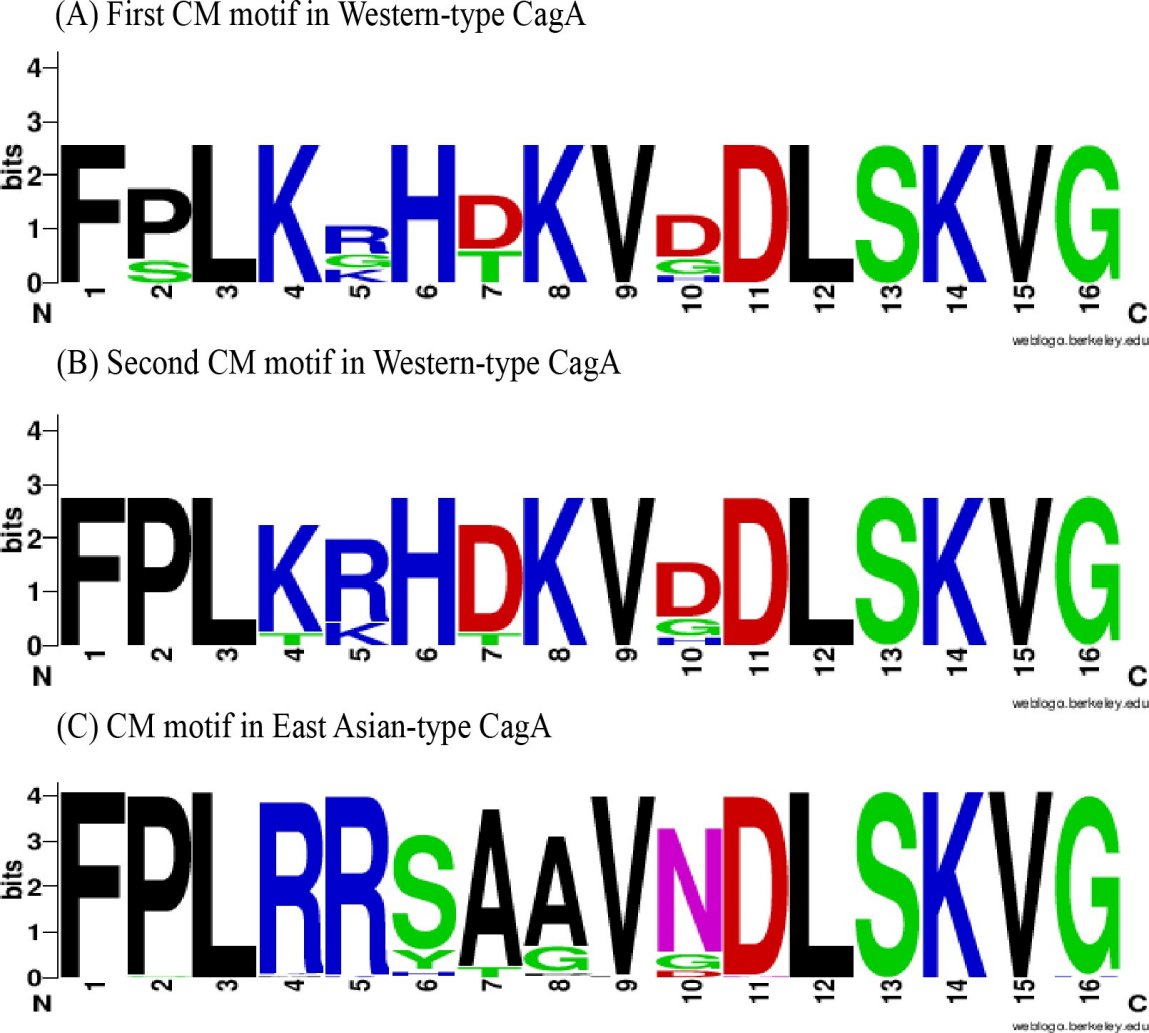
The CM motifs in Western-type and East Asian-type CagA from Shandong strains.

In total, 100 CM motifs were found in 87 *cagA*-positive strains (11 for the 1^st^ CM motif, 88 for the 2^nd^ CM motif, and 1 for the 3^rd^ CM motif). These CM motifs were classified into 28 types, encompassing 8 types in the 1^st^ CM motif, 24 in the 2^nd^ CM motif, and 1 in the 3^rd^ CM motif (Table 7). As shown in Fig 2, the most common Western CM motif type was FPLKRHDKVDDLSKVG, and the most common East Asian CM motif type was FPLRRSAAVNDLSKVG. In this study, sequences demonstrating four or more matching positions with the typical W-CM were designated W-CM motifs, and similarly, those with four or more matching positions with the typical E-CM were designated E-CM motifs. Other sequence types were categorized as different CM (D-CM) motifs. As shown in Table 7, in the 1^st^ CM motif, 8 strains were W-CM, and 3 were D-CM. In the 2^nd^ CM motif, 62 strains were E-CM, 11 were W-CM, and 15 were D-CM. In the 3^rd^ CM motif, 1 strain was W-CM. The combinations of the 1^st^, 2^nd^, and 3^rd^ CM motifs are shown in Table 8, with the three most common CM motif patterns being E (n = 58), followed by D (n = 14), and W-W (n = 5). Next, we analyzed the associations between CM motif patterns and clinical outcomes (Table 9). The CM pattern E was present in 68.66%, 80%, and 40% of the strains isolated from patients with CG, PUD, and GC, respectively. However, the difference was not significant (χ^2^ = 4.119, *P* > 0.05). Conversely, the CM pattern D was significantly more common in strains from GC (50%) patients than in those from CG patients (13.43%) (χ^2^ = 7.821, *P* < 0.01). W-W pattern was present in patients diagnosed with GC. These findings suggested that CagA proteins containing D-CM motifs or multiple W-CM motifs were more virulent than those containing other CM motifs.

**Table 7:** Peptide sequences and types of CM motif in *H*. *pylori* strains.

First motif			Second motif			Third motif		
Peptide sequences	Type	No.	Peptide sequences	Type	No.	Peptide sequences	Type	No.
FPLKRHDKVGDLSKVG	W-CM	2	FPLRRYAPVDDLSKVG	D-CM	2	FPLKRHDKVDDLSKVG	W-CM	1
FPLKKHDKVGDLSKVG	W-CM	1	FPLKKHDKVGDLSKVG	W-CM	2			
FSLKGHTKVDDLSKVG	D-CM	2	FPLRRSAAVNDLSKVG	E-CM	48			
FPLKRHDKVHDLSKVG	W-CM	1	FPLRRSTAVNDLSKVG	E-CM	4			
MKRHDKVDDLSKVG	W-CM	2	FPLRRSAAVNDLSKVR	E-CM	1			
FPLKKHDKVDDLSKVG	W-CM	1	FPLKRHDKVDDLSKVG	W-CM	7			
FPLKRHTKVDDLSKVG	D-CM	1	FPLMRSTAVNDLSKVG	E-CM	1			
FPLKRHDKVDDLSKVG	W-CM	1	FPLRRHAAVNDLSKVG	E-CM	1			
			FPLRRSAAVGNLSKVG	E-CM	1			
			FPLRRSATVNDLSKVG	E-CM	1			
			FPLRRYAGFDDLSKVG	D-CM	1			
			FPLRRSAGVNDLSKVG	E-CM	3			
			FPLKRHDKVHDLSKVG	W-CM	1			
			FPLRRYAAVGDLSKVG	D-CM	1			
			FPLRRYAAVNDLSKVG	E-CM	1			
			FPLRRAAAVNDLSKVG	E-CM	1			
			FPLRRSAGVGDLSKVG	D-CM	3			
			FPLKRHDKVGDLSKVG	W-CM	1			
			FPLRRHAGVGDLSKVG	D-CM	2			
			FPLRKYAGVDDLSKVG	D-CM	1			
			FPLRRYAGVNDLSKVG	D-CM	1			
			FPLRRYAGVGDLSKVG	D-CM	2			
			FPLTRHTKVDDLSKVG	D-CM	1			
			FSLKRYAGVNDLSKVG	D-CM	1			
Total		11	Total		88	Total		1

**Table 8:** CM motif patterns in *H*. *pylori* strains.

CM motif pattern	Occurrence (n = 87)	Frequency (%)
E	58	66.67
D	14	16.09
W-W	5	5.75
W	4	4.6
W-E	2	2.3
D-E	1	1.15
D-W	1	1.15
W-D	1	1.15
W-W-W	1	1.15

CM motif patterns were determined according to the identification of CM sequences that were located before and after the EPIYA-C or EPIYA-D motifs. E: Strains with one East Asian CM motif in their CagA; D: Strains with one Different CM motif in their CagA; W-W: Strains with two Western CM motifs in their CagA; W: Strains with one Western CM motif in their CagA; W-E: Strains with one Western and one East Asian CM motif in their CagA; D-E: Strains with one Different and one East Asian CM motif in their CagA; D-W: Strains with one Different and one Western CM motif in their CagA; W-D: Strains with one Western and one Different CM motif in their CagA; W-W-W: Strains with three Western CM motifs in their CagA.

**Table 9:** Association between CM motif patterns and clinical outcomes.

CM motif pattern	No. of isolates			
	CG (67)	PUD (10)	GC (10)	Total (n = 87)
E	46 (68.66)	8 (80)	4 (40)	58 (66.67)
D	9 (13.43)	0 (0)	**5 (50)** ^a^	14 (16.09)
W-W	4 (5.97)	1 (10)	0 (0)	5 (5.75)
W	4 (5.97)	0 (0)	0 (0)	4 (4.60)
W-E	2 (2.99)	0 (0)	0 (0)	2 (2.30)
D-E	1 (1.49)	0 (0)	0 (0)	1 (1.15)
D-W	0 (0)	1 (10)	0 (0)	1 (1.15)
W-D	1 (1.49)	0 (0)	0 (0)	1 (1.15)
W-W-W	0 (0)	0 (0)	1 (10)	1 (1.15)

CG: chronic gastritis; PUD: peptic ulcer disease; GC: gastric cancer. Values in parentheses are percentages. ^a^ Boldface data indicate a significant difference.

## Discussion

The enduring and intricate coexistence of *H*. *pylori* with humans is notable, with specific bacterial lineages intricately linked to human lineages in specific regions [48]. The genomic structure of *H*. *pylori* exhibits a remarkable degree of variability, revealing substantial differences among different strains. This high variability and polymorphism resulting from genetic mutations account for the variations in the virulence and pathogenicity of *H*. *pylori* [49]. Therefore, identifying the genetic characteristics and polymorphisms of *H*. *pylori* virulence factors is essential for identifying correlations between virulence gene profiles and gastrointestinal diseases. In this study, we determined the prevalence of the *cagA* and *vacA* genotypes in 87 *H*. *pylori* isolates from Shandong and explored their associations with clinical outcomes. These findings highlight significant associations between specific virulence factors and various clinical outcomes, emphasizing the pivotal role of these factors in the development of diseases.

There are large variations in the prevalence of *H*. *pylori* infection worldwide, which can vary depending on various factors, such as socioeconomic status, access to healthcare, and hygiene conditions. The global infection rate of *H*. *pylori* is approximately 50%, and the infection rates in some populations reach 80%-90%. *H*. *pylori* infection is widespread in developing countries, with the highest rate of 80% or more among adults in Africa [50]. America has a relatively low prevalence of *H*. *pylori* infection, and the prevalence is declining in Western countries because of better healthcare and improved living conditions [51]. In recent years, with economic development, improvements in sanitation, and the implementation of public health measures, the prevalence of *H*. *pylori* in China has been declining. In the present study, a relatively low *H*. *pylori* positivity rate of 28.52% was observed. According to the latest estimates, the prevalence of *H*. *pylori* infection in mainland China is 44.2%. In different geographical regions of China, the prevalence of *H*. *pylori* was also evaluated, and the highest rate was 66.4% in Xizang, followed by Gansu (57.2%) and Hebei (52.4%). In contrast, Chongqing, Tianjin, Hunan, and Jilin had the lowest prevalence rates of 35.4%, 36.3%, 37.0%, and 37.6%, respectively [1].

It is widely believed that *cagA* is one of the most important virulence factors intricately linked to increased susceptibility to CG, PUD, precancerous lesions, and GC [52, 53]. The prevalence of *cagA* ranges from 50% to 70% in Western countries [54, 55]; in contrast, almost all *H*. *pylori* strains in East Asian countries carry the *cagA* gene, which may be attributed to the higher incidence rate of GC in East Asian countries than in Western countries [1]. The prevalence of the *cagA* gene in the present study aligned with findings in other Asian countries and some regions of China where the prevalence of *cagA*-positive strains was above 90% [56–58]. Moreover, our investigation revealed a predominance of the East Asian-type *cagA*, with only 12.64% exhibiting the Western-type *cagA*. While previous studies in Japan, Spain, Iraq, and Turkey have demonstrated the association of *cagA* strains with GC or PUD [59–62], our study did not reveal any associations between the *cagA* genotypes and clinical outcomes, which was consistent with our previous study [40].

In the present study, no significant differences were observed between the frequencies of the *vacA* s1 genotype and clinical outcomes, which was inconsistent with previous studies where *vacA* s1 was related to an increased risk of both GC and PUD [36, 63]. Notably, the prevalence of the *vacA* m1 genotype was 60%, 26.87%, and 20% in patients with GC, CG, and PUD, respectively. Statistical analysis revealed a noteworthy association between the *vacA* m1 genotype and GC (OR = 4.275), whereas no such association was observed for CG or PUD. These results were in agreement with those of previous studies from Portugal, America, and the Netherlands [33, 44, 45, 64] but differed from those of other reports that identified an association between the *vacA* m1 genotype and PUD [36, 65], suggesting differences between strains from different regions. The *vacA* i-region, recognized as a key determinant in vacuole-creating activity, has been implicated in the risk for GC and PUD [34, 38, 66]. In contrast to studies in Western countries [44, 67, 68], the present study did not reveal any associations between the *vacA* i1 genotype and clinical outcomes. With respect to the d-region, the *vacA* d1 genotype, when associated with the s1, m1, and i1 genotypes, demonstrated an increased risk of GC [44]. Additionally, studies have shown that the *vacA* d1 genotype is significantly associated with GC [32, 45] and PUD [69]. In the present study, the frequency of the *vacA* d1 genotype was greater in patients with PUD and GC (90%) than in those with CG (86.57%), but the difference did not reach significance. Notably, the *vacA* c1 genotype was significantly associated with the risk of GC in Shandong (*P* < 0.05). Statistical analysis revealed that the frequency of the *vacA* c1 genotype in GC patients (50%) was greater than that in CG patients (19.4%), yielding an OR of 4.923. Additionally, the combined presence of the *vacA* m1 and c1 genotypes, along with the *vacA* i1 and d1 genotypes, further increased susceptibility to GC, with the *vacA* m1i1c1 genotype combination showing the highest risk (OR = 5.417). Despite these associations, the biological role of this genotype combination in terms of vacuolar production activity and disease incidence remains unclear. In summary, simple logistic regression model analyses demonstrated a significant association of the *vacA* m1 and c1 genotypes with the risk of GC, whereas the s1, i1, d1, and *cagA* genotypes did not exhibit such associations. However, multivariate analysis revealed that the *vacA* c1 genotype was most strongly associated with an increased risk of GC, with an OR of 5.174. Statistical analysis revealed a significant association in male patients with GC aged ≥55 years (OR = 4.386).

We confirmed the prevalent presence of the East Asian-type CagA in the *H*. *pylori* strains from Shandong, demonstrating an association with more severe gastric mucosal inflammation and a greater incidence of GC than the Western-type CagA. Among the East Asian-type CagA strains in Shandong, the CagA ABD type was predominant (90.79%). Previous studies have indicated a greater incidence of PUD and GC in patients infected with strains carrying multiple EPIYA-C segments than in those with a single segment [70, 71]. However, in our study, only one CagA sequence contained two EPIYA-C segments, and no significant correlation was detected between the number of EPIYA-C segments in the Western-type CagA strains and clinical outcomes. Additionally, single amino acid variations in EPIYA motifs exhibited differences, suggesting a potential role in the varying virulence of *H*. *pylori* strains. In Shandong, EPIYA (92.4%) was the predominant type, followed by EPIYT (5.2%) and ESIYA (2.16%). Previous studies reported that the EPIYA-B motif displayed the greatest variation in the Western-type CagA but was very rare in the East Asian-type CagA [72, 73]. Our previous study of 1,587 EPIYA motifs from 503 CagA strains revealed that 92.1% were EPIYA, followed by EPIYT (4.7%) and ESIYA (1.4%) [47]. Zhang et al. reported that there was a significant correlation between EPIYT sequences and GC [72]. The role of EPIYT sequences in the pathogenesis of *H*. *pylori*-associated disease needs further study.

The variations in Western (W-) and East Asian (E-) CM motifs and their correlation with clinical outcomes remain underexplored in Shandong. Our analysis revealed a substantial degree of heterogeneity in the arrangement of W- and E-CM motifs. Twenty-eight kinds of CM motifs were observed in 87 *cagA*-positive strains, which was greater in number than those in Myanmar, Thailand, and Bangladesh [74–76]. Conversely, the number of CM motifs in Shandong was comparable to findings in Colombia and America [25, 26]. The CM motif pattern with the greatest number of occurrences was E, followed by D and W-W. The analysis of the CM motif patterns in our study suggested that the CM pattern D was significantly greater in strains from GC patients than in those from CG patients, which was inconsistent with the findings of previous studies [25, 26]. Research has shown that CagA proteins with two or more W-CM motifs were associated with more severe gastric disorders, such as PUD and GC, which was consistent with our findings that the W-W pattern was present only in patients diagnosed with GC [25]. Consequently, the types and patterns of CM motifs may play crucial roles in the pathogenesis of *H*. *pylori*-associated diseases.

This report is the first study to elucidate in detail the prevalence of *cagA* and *vacA* genotypes, along with a comprehensive analysis of the characteristics of the CagA EPIYA and CM motif types in Shandong. Despite the valuable insights obtained, certain limitations necessitate consideration. First, the sample size of strains isolated from GC patients was limited, potentially impacting the generalizability of our findings. Second, our study focused on only two specific regions within Shandong, so our results may not fully encapsulate the entire spectrum of virulence factor distributions in Shandong, given the evident regional variations. Third, the number of female patients was small in the process of collecting gastric mucosa samples, resulting in an unbalanced proportion of male and female patients. Future investigations should aim to overcome these limitations by incorporating a larger sample size encompassing diverse gastrointestinal diseases in various regions within Shandong and ensuring that the sample collected is representative and can accurately reflect the characteristics of the population being studied.

## Conclusions

In conclusion, we found that CagA proteins possessing CM motif pattern D were observed more frequently in patients with GC, and the profiling of diverse CM motifs has emerged as a potentially valuable tool for assessing the toxicity of *H*. *pylori* and predicting *H*. *pylori*-related diseases. Furthermore, a robust association was identified between the *vacA* c1 genotype and GC. We speculate that the *vacA* c1 genotype may serve as a particularly potent risk indicator for GC, particularly among male patients aged ≥55 years in Shandong. However, further studies are imperative to substantiate our hypothesis and enhance the understanding of these intricate associations.

## Supporting information

S1 Table Frequency of 87 *H*. *pylori* virulence genotypes based on the distribution of age, sex, and disease. CSG: chronic superficial gastritis; CAG: chronic atrophic gastritis; PUD: peptic ulcer disease; GC: gastric cancer. Values in parentheses are percentages.
(DOCX)
