# Supplementary material for: Virulence gene polymorphisms in Shandong Helicobacter pylori strains and their relevance to gastric cancer
Source: PLoS One. 2024 Sep 9;19(9):e0309844. doi: 10.1371/journal.pone.0309844 (PMC11383249; doi:10.1371/journal.pone.0309844)
Supplement: S1 Table — CSG: chronic superficial gastritis; CAG: chronic atrophic gastritis; PUD: peptic ulcer disease; GC: gastric cancer. Values in parentheses are percentages. (DOCX) [file pone.0309844.s001.docx]

S1 Table Distribution of 87 *H. pylori* virulence genotypes based on age, sex, and disease

| Genotypes | No. of isolates | No. of isolates | | | | | | | |
| --- | --- | --- | --- | --- | --- | --- | --- | --- | --- |
|  |  | Age groups | | Sex groups | | Disease groups | | | |
|  |  | ≥55 years | <55 years | males | females | CSG | CAG | PUD | GC |
| *cagA* | 87(100) |  |  |  |  |  |  |  |  |
| East Asian-type *cagA* | 76(87.36) | 44(57.89) | 32(42.11) | 54(71.05) | 22(28.95) | 13(17.11) | 46(60.53) | 8(80) | 9(90) |
| Western-type *cagA* | 11(12.64) | 5(45.45) | 6(54.55) | 8(72.73) | 3(27.27) | 3(27.27) | 5(45.45) | 2(20) | 1(10) |
| *vacA* | 87(100) |  |  |  |  |  |  |  |  |
| s1 | 87(100) | 49(56.32) | 38(43.68) | 62(71.26) | 25(28.74) | 16(18.39) | 51(58.62) | 10(100) | 10(100) |
| s2 | 0(0) | 0(0) | 0(0) | 0(0) | 0(0) | 0(0) | 0(0) | 0(0) | 0(0) |
| m1 | 26(29.89) | 14(53.85) | 12(46.15) | 18(69.23) | 8(30.77) | 5(19.23) | 13(50) | 2(20) | 6(60) |
| m2 | 61(70.11) | 35(57.38) | 26(42.62) | 44(72.13) | 17(27.87) | 11(18.03) | 38(62.30) | 8(80) | 4(40) |
| i1 | 73(83.91) | 43(58.90) | 30(41.10) | 54(73.97) | 19(26.03) | 12(16.44) | 45(61.64) | 8(80) | 8(80) |
| i2 | 14(16.09) | 6(42.86) | 8(57.14) | 8(57.14) | 6(42.86) | 4(28.57) | 6(42.86) | 2(20) | 2(20) |
| d1 | 76(87.36) | 44(57.89) | 32(42.11) | 54(71.05) | 22(28.95) | 14(18.42) | 44(57.89) | 9(90) | 9(90) |
| d2 | 11(12.64) | 5(45.45) | 6(54.55) | 8(72.73) | 3(27.27) | 2(18.18) | 7(63.64) | 1(10) | 1(10) |
| c1 | 18(20.69) | 11(61.11) | 7(38.89) | 14(77.78) | 4(22.22) | 4(22.22) | 9(50) | 0(0) | 5(50) |
| c2 | 69(79.31) | 38(55.07) | 31(44.93) | 48(69.57) | 21(30.43) | 12(17.39) | 42(60.87) | 10(100) | 5(50) |
| m1i1 | 26(29.89) | 14(53.85) | 12(46.15) | 18(69.23) | 8(30.77) | 5(19.23) | 13(50) | 2(20) | 6(60) |
| m2i2 | 14(16.09) | 6(42.86) | 8(57.14) | 8(57.14) | 6(42.86) | 4(28.57) | 6(42.86) | 2(20) | 2(20) |
| m1d1 | 26(29.89) | 14(53.85) | 11(42.31) | 17(65.38) | 8(30.77) | 5(19.23) | 13(50) | 2(20) | 6(60) |
| m2d2 | 11(12.64) | 5(45.45) | 6(54.55) | 8(72.73) | 3(27.27) | 2(18.18) | 7(63.64) | 1(10) | 1(10) |
| m1c1 | 18(20.69) | 11(61.11) | 7(38.89) | 14(77.78) | 4(22.22) | 4(22.22) | 9(50) | 0(0) | 5(50) |
| m2c2 | 61(70.11) | 34(55.74) | 27(44.26) | 43(70.49) | 17(27.87) | 11(18.03) | 38(62.30) | 8(80) | 4(40) |
| i1d1 | 70(80.46) | 41(58.57) | 29(41.43) | 52(74.29) | 18(25.71) | 12(17.14) | 42(60) | 8(80) | 8(80) |
| i2d2 | 8(9.20) | 3(37.50) | 5(62.50) | 6(75) | 2(25) | 2(25) | 4(50) | 1(10) | 1(10) |
| i1c1 | 18(20.69) | 11(61.11) | 7(38.89) | 14(77.78) | 4(22.22) | 4(22.22) | 9(50) | 0(0) | 5(50) |
| i2c2 | 14(16.09) | 6(42.86) | 8(57.14) | 8(57.14) | 6(42.86) | 4(28.57) | 6(42.86) | 4(40) | 0(0) |
| d1c1 | 18(20.69) | 11(61.11) | 7(38.89) | 14(77.78) | 4(22.22) | 4(22.22) | 9(50) | 0(0) | 5(50) |
| d2c2 | 11(12.64) | 5(45.45) | 6(54.55) | 8(72.73) | 3(27.27) | 2(18.18) | 7(63.64) | 1(10) | 1(10) |
| m1i1d1 | 26(29.89) | 14(53.85) | 12(46.15) | 18(69.23) | 8(30.77) | 5(19.23) | 13(50) | 2(20) | 6(60) |
| m2i2d2 | 8(9.20) | 3(37.50) | 5(62.50) | 6(75) | 2(25) | 2(25) | 4(50) | 1(10) | 1(10) |
| m1i1c1 | 18(20.69) | 10(55.56) | 7(38.89) | 13(72.22) | 4(22.22) | 5(27.78) | 8(44.44) | 0(0) | 5(50) |
| m2i2c2 | 14(16.09) | 6(42.86) | 8(57.14) | 8(57.14) | 6(42.86) | 4(28.57) | 6(42.86) | 2(20) | 2(20) |
| m1d1c1 | 18(20.69) | 11(61.11) | 7(38.89) | 14(77.78) | 4(22.22) | 4(22.22) | 9(50) | 0(0) | 5(50) |
| m2d2c2 | 11(12.64) | 5(45.45) | 6(54.55) | 8(72.73) | 3(27.27) | 2(18.18) | 7(63.64) | 1(10) | 1(10) |
| i1d1c1 | 18(20.69) | 11(61.11) | 7(38.89) | 14(77.78) | 4(22.22) | 4(22.22) | 9(50) | 0(0) | 5(50) |
| i2d2c2 | 8(9.20) | 3(37.50) | 5(62.50) | 6(75) | 2(25) | 2(25) | 4(50) | 1(10) | 1(10) |
| m1i1d1c1 | 18(20.69) | 11(61.11) | 7(38.89) | 14(77.78) | 4(22.22) | 4(22.22) | 9(50) | 0(0) | 5(50) |
| m2i2d2c2 | 8(9.20) | 3(37.50) | 5(62.50) | 6(75) | 2(25) | 2(25) | 4(50) | 1(10) | 1(10) |
| s1m2i1d1c2 | 44(50.57) | 27(61.36) | 17(38.64) | 34(77.27) | 10(22.73) | 7(15.91) | 29(65.91) | 6(60) | 2(20) |
| s1m1i1d1c1 | 18(20.69) | 11(61.11) | 7(38.89) | 14(77.78) | 4(22.22) | 4(22.22) | 9(50) | 0(0) | 5(50) |
| s1m1i1d1c2 | 8(9.20) | 3(37.50) | 5(62.50) | 4(50) | 4(50) | 1(12.50) | 4(50) | 2(20) | 1(10) |
| s1m2i2d2c2 | 8(9.20) | 3(37.50) | 5(62.50) | 6(75) | 2(25) | 2(25) | 4(50) | 1(10) | 1(10) |
| s1m2i2d1c2 | 6(6.90) | 3(50) | 3(50) | 2(33.33) | 4(66.67) | 2(33.33) | 2(33.33) | 1(10) | 1(10) |
| s1m2i1d2c2 | 3(3.45) | 2(66.67) | 1(33.33) | 2(66.67) | 1(33.33) | 0(0) | 3(100) | 0(0) | 0(0) |

Values in parentheses are percentages. CSG: chronic superficial gastritis, CAG: chronic atrophic gastritis, PUD: peptic ulcer disease, GC: gastric cancer.
